# Genetic causes of hypercalciuric nephrolithiasis

**DOI:** 10.1007/s00467-008-0807-0

**Published:** 2008-04-30

**Authors:** Michael J. Stechman, Nellie Y. Loh, Rajesh V. Thakker

**Affiliations:** Academic Endocrine Unit, Nuffield Department of Clinical Medicine, Oxford Centre for Diabetes, Endocrinology and Metabolism (OCDEM), University of Oxford, Oxford, OX3 7LJ UK

**Keywords:** Nephrolithiasis, Nephrocalcinosis, Genetic hypercalciuria, Calcium, Magnesium, Phosphate, Inheritance, Hereditary, Renal tubular disorders

## Abstract

Renal stone disease (nephrolithiasis) affects 3–5% of the population and is often associated with hypercalciuria. Hypercalciuric nephrolithiasis is a familial disorder in over 35% of patients and may occur as a monogenic disorder that is more likely to manifest itself in childhood. Studies of these monogenic forms of hypercalciuric nephrolithiasis in humans, e.g. Bartter syndrome, Dent’s disease, autosomal dominant hypocalcemic hypercalciuria (ADHH), hypercalciuric nephrolithiasis with hypophosphatemia, and familial hypomagnesemia with hypercalciuria have helped to identify a number of transporters, channels and receptors that are involved in regulating the renal tubular reabsorption of calcium. Thus, Bartter syndrome, an autosomal disease, is caused by mutations of the bumetanide-sensitive Na–K–Cl (NKCC2) co-transporter, the renal outer-medullary potassium (ROMK) channel, the voltage-gated chloride channel, CLC-Kb, the CLC-Kb beta subunit, barttin, or the calcium-sensing receptor (CaSR). Dent’s disease, an X-linked disorder characterized by low molecular weight proteinuria, hypercalciuria and nephrolithiasis, is due to mutations of the chloride/proton antiporter 5, CLC-5; ADHH is associated with activating mutations of the CaSR, which is a G-protein-coupled receptor; hypophosphatemic hypercalciuric nephrolithiasis associated with rickets is due to mutations in the type 2c sodium–phosphate co-transporter (NPT2c); and familial hypomagnesemia with hypercalciuria is due to mutations of paracellin-1, which is a member of the claudin family of membrane proteins that form the intercellular tight junction barrier in a variety of epithelia. These studies have provided valuable insights into the renal tubular pathways that regulate calcium reabsorption and predispose to hypercalciuria and nephrolithiasis.

## Introduction

Nephrolithiasis (kidney stone disease), which is a common disorder that affects approximately 3–5% of the population [1], is usually associated with a metabolic abnormality that may include hypercalciuria, hyperphosphaturia, hyperoxaluria, hypocitraturia, hyperuricosuria, cystinuria, a low urinary volume and a defect in urinary acidification [2, 3]. If uncorrected, these metabolic abnormalities may lead to recurrent stone formation, whose rate may be as high as 50% at 5 years [4, 5]. The etiology of these metabolic abnormalities and of renal stones is multi-factorial and involves interactions between environmental and genetic determinants [6–8]. The environmental determinants include dietary intake of salt, protein, calcium and other nutrients, fluid intake, urinary tract infections, socio-economic status of the individual, lifestyle and climate [9, 10]. This review will focus on the progress made on the genetic determinants of hypercalciuric nephrolithiasis (Table 1), as these are more likely to present in childhood.

**Table 1:** Genetic defects associated with some monogenic forms of hypercalciuria

Disease^a^	Mode of inheritance^b^	Gene^c^	Human chromosomal location	Reference
Idiopathic hypercalciuria				
	A-d	SAC	1q23.3-q24	[35]
	A-d	VDR	12q12-q14	[30]
	A-d	?	9q33.2-q34.2	[31]
ADHH	A-d	CASR	3q21.1	[40]
Hypercalcemia with hypercalciuria	A-d	CASR	3q21.1	[38]
Bartter syndromes				
Type I	A-r	SLC12A1/NKCC2	15q15-q21.1	[90]
Type II	A-r	KCNJ1/ROMK	11q24	[91]
Type III^d^	A-r	CLCNKB	1q36	[92]
Type IV^d^	A-r	BSND	1q31	[93]
Type V	A-d	CASR	3q21.1	[48]
Type VI^e^	X-r	CLCN5	Xp11.22	[50]
Dent’s disease	X-r	CLCN5	Xp11.22	[52]
Lowe’s syndrome	X-r	OCRL1	Xq25	[60]
HHRH	A-r	NPT2c/SLC34A3	9q34	[69]
Nephrolithiasis, osteoporosis and hypophosphatemia	A-d	NPT2a/SLC34A1	5q35	[64]
Familial hypomagnesemia with hypercalciuria and nephrocalcinosis	A-r	PCLN1/CLDN16	3q28	[73]
Familial hypomagnesemia with hypercalciuria and nephrocalcinosis with ocular abnormalities	A-r	CLDN19	1p34.2	[77]
dRTA	A-d	SLC4A1/kAE1	17q21.31	[81]
dRTA with sensorineural deafness	A-r	ATP6B1/ATP6V1B1	2p13	[85]
dRTA with preserved hearing	A-r	ATP6N1B/ATP6V0A4	7q34	[88]

^a^*ADHH* autosomal dominant hypocalcemia with hypercalciuria, *HHRH* hereditary hypophosphatemic rickets with hypercalciuria, dRTA distal renal tubular acidosis

^b^*A-d* autosomal dominant, *A-r* autosomal recessive, *X-r* X-linked recessive

^c^*SAC* human soluble adenylyl cyclase; *VDR* vitamin D receptor; *CASR* calcium-sensing receptor; *SLC12A1* solute carrier family 12, member 1; *NKCC2* sodium–potassium–chloride co-transporter 2; *KCNJ1* potassium channel, inwardly rectifying, subfamily J, member 1; *ROMK* renal outer medullary potassium channel; *CLCNKB* chloride channel Kb; *BSND* Barttin; *CLCN5* chloride channel 5; *OCRL1* oculo-cerebro-renal syndrome of Lowe 1; *NPT2c/a* sodium–phosphate co-transporter type 2c/a; *SLC34A1/3* solute carrier family 34, member 1/3; *PCLN1* paracellin; *CLDN16/19* claudin 16/19; *SLC4A1* solute carrier family 4, member 1; *kAE1* kidney anion exchanger 1; *ATP6B1* ATPase, H^+^ transporting (vacuolar proton pump), V1 subunit B1; *ATP6N1B* ATPase, H^+^ transporting, lysosomal V0 subunit a4

^d^Bartter type III is associated with hypercalciuria but not nephrocalcinosis; Bartter type IV is not associated with persistent hypercalciuria or nephrocalcinosis, but these are included here for completeness

^e^Bartter type VI has been reported in one patient only, who had hypercalciuria

Nephrolithiasis may affect children of all ages, with preponderance for boys [11], but it is important to note that children represent only 2–3% of the total population of stone formers [12]. However, nephrolithiasis is associated with a metabolic abnormality in approximately 50% of children. The younger child will likely present with hematuria or urinary tract infection due to stones sited predominantly in the kidney, whilst the adolescent child is more likely to develop ureteric stones and classical renal colic [13]. More than 75% of these children will have stones composed of calcium phosphate or calcium oxalate [14], approximately 25% will have an associated urinary tract infection [15, 16] and 30% will have anatomical abnormalities that include hydronephrosis, posterior urethral valves and fused crossed renal ectopy [14]. The affected children are at risk of chronic urinary tract infections and progressive kidney disease, but treatment of the metabolic abnormality and nephrolithiasis will help to prevent these complications.

## Genetic epidemiology of nephrolithiasis and hypercalciuria

The greatest risk factor for nephrolithiasis, after controlling for known dietary determinants, is having an affected family member [17], and it has been known since the 1890s that patients with renal stones are more likely than those that are not renal stone formers to have other family members affected with nephrolithiasis [18]. Thus, between 35% and 65% of renal stone formers will have relatives with nephrolithiasis, whereas only 5–20% of those that are not renal stone formers will have relatives with nephrolithiasis [17, 19, 20]. The first degree relative risk (λ _R_) amongst recurrent stone formers has been estimated to be between 2 and 16 [17, 21, 22]. The wide range of these estimates is largely due to differences in the study designs and the methods used to ascertain the occurrence of renal stones in relatives. Moreover, studies of twins have shown that the contributions of heritability to kidney stones and urinary calcium excretion are as high as 56% and 52%, respectively [23, 24].

Hypercalciuria, which is defined as a urinary calcium excretion in excess of 0.1 mmol/kg per 24 h or 4 mg/kg per 24 h [25, 26] is the most common metabolic abnormality associated with nephrolithiasis and is found in approximately 60% of patients with renal stones [2, 27]. Hypercalciuria may occur either as an isolated trait or in association with other metabolic abnormalities, and also as part of a renal tubular disorder. Up to 65% of patients with hypercalciuric nephrolithiasis may have a family history of the disorder [17, 19] and the inheritance may occur as that of a polygenic quantitative trait, or of a monogenic trait with either autosomal dominant, autosomal recessive or X-linked recessive modes of transmission (Table 1). The monogenic forms of hypercalciuric nephrolithiasis and their associated underlying molecular genetic mechanisms that may be associated with defects of intestinal calcium absorption, bone calcium resorption, and renal calcium reabsorption will be reviewed (Table 1).

## Monogenic forms of hypercalciuria with nephrolithiasis

### Idiopathic hypercalciuria

Families with idiopathic hypercalciuria (IH) and recurrent calcium oxalate stones usually reveal an autosomal dominant mode of inheritance [28]. Studies of such families have established linkage between hypercalciuric nephrolithiasis and loci on: chromosome 1q23.3-q24, which contains the human soluble adenylyl cyclase (SAC) gene [29]; chromosome 12q12-q14, which contains the vitamin D receptor (VDR) gene [30]; and chromosome 9q33.2-q34.2, from which an appropriate candidate gene remains to be identified [31].

#### Absorptive hypercalciuria locus on 1q23.3-q24

Absorptive hypercalciuria (AH), which is characterized by increased intestinal calcium uptake, normocalcemia, normal concentrations of circulating parathyroid hormone (PTH), and low bone mineral density [32], may occur as an autosomal dominant trait [33]. Linkage studies in three families with AH, mapped the locus to chromosome 1q23.3-q24 [29] and to a region that contained a gene encoding a human SAC, which is a divalent cation and bicarbonate sensor [34]. The SAC protein, which does not respond to the heterotrimeric G protein regulators, exists freely in cytosolic and membrane-associated forms, and its cyclase catalytic activity helps to facilitate the generation of cyclic adenosine monophosphate (cAMP) in the vicinity of its targets. Mutational analysis of SAC in patients with AH revealed six sequence variations, and four of these were shown to be associated with a significantly increased relative risk for AH [35]. The manner in which these four sequence variations of SAC result in hypercalciuria and nephrolithiasis remains to be elucidated.

#### Vitamin D receptor and IH

Increased levels of VDR are found in circulating monocytes from patients with IH [36], and studies in a cohort of large French-Canadian families have revealed an association between IH nephrolithiasis and polymorphic loci from chromosome 12q12-q14, a region that contains the VDR gene [30]. However, DNA sequence analysis of the VDR gene did not identify any VDR mutations, but it did reveal conservative substitutions within the coding region. The role of these VDR polymorphisms in the etiology of IH nephrolithiasis remains to be explained.

#### Chromosome 9q33.2-q34.2 locus for autosomal dominant nephrolithiasis

A form of autosomal dominant nephrolithiasis (NPL1) has been reported in a Spanish kindred that originated from La Gomera, in the Canary Islands, and resides in Tenerife [31]. The renal stones were reported to consist of calcium oxalate, and serum and urine analyses did not reveal any significant abnormalities, although some affected members had mild hypercalciuria and some had hypomagnesemia. Linkage analysis mapped the NPL1 locus to chromosome 9q33.2-q34.2. This region contains approximately 170 genes, and, to date, the gene causing NPL1 has not been identified [31].

### Calcium-sensing receptor and hypercalciuric disorders

The human calcium-sensing receptor (CaSR) is a 1,078 amino acid cell surface protein, which is predominantly expressed in the parathyroids and kidney and is a member of the family of G protein-coupled receptors. The CaSR allows regulation of PTH secretion and renal tubular calcium reabsorption in response to alterations in extracellular calcium concentrations. The human CASR gene is located on chromosome 3q21.1, and loss-of-function CaSR mutations have been reported in the hypercalcemic disorders of familial benign (hypocalciuric) hypercalcemia (FBHH), neonatal severe primary hyperparathyroidism (NSHPT) and familial isolated hyperparathyroidism (FIHP). However, gain-of-function CaSR mutations result in autosomal dominant hypocalcemia with hypercalciuria (ADHH) and Bartter’s syndrome type V.

#### Familial isolated primary hyperparathyroidism due to CaSR mutations

Hereditary disorders associated with hypercalcemia include familial isolated primary hyperparathyroidism (FIHP), familial benign hypocalciuric hypercalcemia (FBHH), multiple endocrine neoplasia type 1 (MEN1), MEN type 2 (MEN2), and the hyperparathyroidism–jaw tumor (HPT-JT) syndrome. The hypercalcemia of FIHP, MEN1, MEN2, and HPT-JT (Table 2) is associated with hypercalciuria and sometimes kidney stones, whereas that of FBHH is associated with a low urinary calcium–creatinine clearance ratio (< 0.01). In FBHH, which is an autosomal dominant disorder, the hypercalcemia is usually mild to moderate, i.e. within 10% of the upper limit of normal, although some patients do have more severe hypercalcemia [37]. Other biochemical features include mild hypermagnesemia and normal or mildly elevated serum PTH concentrations. FBHH is due to inactivating mutations of the CASR gene, which is located on chromosome 3q21.1, whereas the genes causing MEN1, MEN2 and HPT-JT are located on chromosomes 11q13, 10q11.2, and 1q31.2, respectively. Some patients with FIHP have mutations of the MEN1, HPT-JT and CaSR genes, although the majority of FIHP patients do not have such mutations and the genes involved need to be characterized. Five CaSR mutations (Thr100Ile, Lys336deletion, Leu650Pro, Val689Met and Phe881Leu) have been reported in FIHP [38, 39]. Functional characterization of the mutant Phe881Leu CaSR only, has been undertaken in HEK293 cells, and this demonstrated that the Phe881Leu mutation resulted in a loss of function [38]. Thus, although the majority of loss-of-function CaSR mutations will lead to FBHH, some may result in hypercalcemic adenoma formation.

**Table 2:** Hereditary diseases associated with hypercalcemia and hypercalciuria (*FIHP* familial isolated hyperparathyroidism, *MEN* multiple endocrine neoplasia, *HPT-JT* hyperparathyroidism–jaw tumor syndrome)

Disorder	Clinical features	Gene product	Chromosomal location of the gene
FIHP	Familial isolated parathyroid tumors	Menin	11q13
		Parafibromin	1q31.2
		CaSR	3q21.1
MEN1	Parathyroid hyperplasia and/or tumors associated with pituitary and pancreatico-duodenal neuro-endocrine tumors	Menin	11q13
MEN2a	Parathyroid tumors with medullary thyroid cancer and pheochromocytoma	Ret	10q11.2
HPT-JT	Parathyroid tumors with ossifying fibromas of the jaw	Parafibromin	1q31.2

#### Autosomal dominant hypocalcemic hypercalciuria

Patients with ADHH usually have mild hypocalcemia that is generally asymptomatic, but it may, in some patients, be associated with carpo-pedal spasm and seizures [40]. The serum phosphate concentrations in patients with ADHH are either elevated or in the upper–normal range, and the serum magnesium concentrations are either low or in the low–normal range [40]. These biochemical features of hypocalcemia, hyperphosphatemia and hypomagnesemia are consistent with hypoparathyroidism and pseudo-hypoparathyroidism. However, these patients have serum PTH concentrations that are in the low–normal range [40–44]. Thus, they are not hypoparathyroid, which would be associated with undetectable serum PTH concentrations, or pseudo-hypoparathyroid, which would be associated with elevated serum PTH concentrations. These patients were therefore classified as having autosomal dominant hypocalcemia (ADH) [41], and the association of hypercalciuria with this condition led to its being referred to as autosomal dominant hypocalcemia with hypercalciuria (ADHH) [40]. Treatment with active metabolites of vitamin D to correct the hypocalcemia has been reported to result in marked hypercalciuria, nephrocalcinosis, nephrolithiasis and renal impairment, which was partially reversible after cessation of the vitamin D treatment [40]. Thus, it is important to identify and restrict the use of vitamin D treatment in such ADHH patients and their families whose hypocalcemia is due to a gain-of-function CaSR mutation and not hypoparathyroidism [40]. More than 40 different CASR mutations have been identified in ADHH patients, and over 50% of these are in the extracellular domain [40–45]. Almost every ADHH family has its own unique missense heterozygous CASR mutation [45]. Studies of the expression of ADHH-associated CaSR mutations have demonstrated a gain-of-function, whereby there is a leftward shift in the dose–response curve, such that the extracellular calcium concentration needed to produce a half-maximal effective concentration (EC_50_) increase in total intracellular calcium ions (or inositol trisphosphate, IP_3_) is significantly lower than that required for the wild-type receptor [40, 41].

#### Bartter syndrome type V

Bartter syndrome is a heterogeneous group of autosomal hereditary disorders of electrolyte homeostasis characterized by hypokalemic alkalosis, renal salt wasting that may lead to hypotension, hyperreninemic hyperaldosteronism, increased urinary prostaglandin excretion, and hypercalciuria with nephrocalcinosis [46, 47]. Mutations of several ion transporters and channels have been associated with Bartter syndrome, and six types (Table 1) are now recognized [47]. Thus, type I is due to mutations involving the bumetanide-sensitive sodium–potassium–chloride co-transporter (NKCC2 or SLC12A1); type II is due to mutations of the renal outer-medullary potassium (ROMK) channel; type III is due to mutations of the voltage-gated chloride channel, CLC-Kb; type IV is due to mutations of barttin, which is a beta sub-unit that is required for trafficking of CLC-Kb and CLC-Ka, and this form is also associated with deafness as barttin, CLC-Ka and CLC-Kb are also expressed in the marginal cells of the scala media of the inner ear that secrete potassium ion-rich endolymph; and type V is due to activating mutations of the CaSR. Patients with Bartter syndrome type V have the classical features of the syndrome, i.e. hypokalemic metabolic alkalosis, hyperreninemia and hyperaldosteronism [48, 49]. In addition, they develop hypocalcemia, which may be symptomatic and lead to carpo-pedal spasm, and an elevated fractional excretion of calcium, that may be associated with nephrocalcinosis [48, 49]. Such patients have been reported to have heterozygous gain-of-function CaSR mutations, and in vitro functional expression of these mutations not only revealed a leftward shift in the dose–response curve for the receptor, but also showed them to have a much lower EC_50_ than that found in patients with ADHH [47–49]. This suggests that the additional features that occur in Bartter syndrome type V when compared to ADHH are due to severe gain-of-function mutations of the CaSR [47]. Bartter syndrome type VI has been reported in one child from Turkey [50] and was associated with a *CLCN5* mutation; the latter are usually seen in Dent’s disease (see below).

### Dent’s disease

Dent’s disease is an X-linked recessive renal tubular disorder characterized by low molecular weight proteinuria, hypercalciuria, nephrocalcinosis, nephrolithiasis, and eventual renal failure [51]. Dent’s disease is also associated with the other multiple proximal tubular defects of the renal Fanconi syndrome, which include aminoaciduria, phosphaturia, glycosuria, kaliuresis, uricosuria, and impaired urinary acidification [51]. With the exception of rickets, which occurs in a minority of patients, there appear to be no extra-renal manifestations in Dent’s disease [51]. The gene causing Dent’s disease, *CLCN5*, encodes the chloride/proton antiporter CLC-5 [52]. CLC family members, which are usually voltage-gated chloride channels, have important diverse functions that include the control of membrane excitability, transepithelial transport and regulation of cell volume [53]. CLC-5, which is predominantly expressed in the kidney and, in particular, the proximal tubule, thick ascending limb of Henle, and the alpha intercalated cells of the collecting duct, has been reported to be critical for acidification in the endosomes that participate in solute re-absorption and membrane recycling in the proximal tubule [54, 55]. CLC-5 is also known to alter membrane trafficking via the receptor-mediated endocytic pathway that involves megalin and cubulin [56]. CLC-5 mutations associated with Dent’s disease impair chloride flow and likely lead to impaired acidification of the endosomal lumen and, thereby, also disrupt trafficking of endosomes back to the apical surface [56]. This will result in impairment of solute reabsorption by the renal tubule and in the defects observed in Dent’s disease [57]. Mice that are deficient for CLC-5 develop the phenotypic abnormalities associated with Dent’s disease [57]. Mutations of the gene encoding an inositol polyphosphate 5-phosphatase result in Lowe syndrome (see below) and also Dent’s disease [58].

### Oculo-cerebro-renal syndrome of Lowe

The oculo-cerebro-renal syndrome of Lowe (OCRL) is an X-linked recessive disorder that is characterized by congenital cataracts, mental retardation, muscle hypotonia, rickets, and defective proximal tubular reabsorption of bicarbonate, phosphate and amino acids. Some patients may also develop hypercalciuria and renal calculi [59]. The disease is nearly always confined to boys, who develop renal dysfunction in the first year of life, have delayed bone age and reduced height, and may die in childhood. Female carriers who have normal neurological and renal function can be identified in 80% of cases by micro-punctate cortical lens opacities. The Lowe syndrome gene, *OCRL1*, is located on Xq25 and encodes a member of the type II family of inositol polyphosphate 5-phosphatases [60]. These enzymes hydrolyze the 5-phosphate of inositol 1,4,5-trisphosphate and of inositol 1,3,4,5-tetrakisphosphate, phosphatidylinositol 4,5-bisphosphate, and phosphatidylinositol 3,4,5-trisphosphate, thereby presumably inactivating them as second messengers in the phosphatidylinositol signaling pathway [61]. The preferred substrate of OCRL1 is phosphatidylinositol 4,5-bisphosphate, and this lipid accumulates in the renal proximal tubular cells of patients with Lowe syndrome [61]. OCRL1 is localized to lysosomes in renal proximal tubular cells and to the trans-Golgi network in fibroblasts. This localization is consistent with the role of OCRL1 in lysosomal enzyme trafficking from the trans-Golgi network to lysosomes, and the activities of several lysosomal hydrolases are found to be elevated in the plasma of affected patients [62]. OCRL1 has also been shown to interact with clathrin and indeed co-localizes with clathrin on endosomal membranes that contain transferrin and mannose 6-phosphate receptors [63]. Mannose 6-phosphate receptor-bound lysosomal enzymes are recruited by appendage (AP) subunits and Golgi-localized binding proteins into clathrin-coated vesicles that transport them from the trans-Golgi network to endosomes [63]. Thus, it seems likely that the *OCRL1* mutations in Lowe syndrome patients result in OCRL1 protein deficiency, which leads to disruptions in lysosomal trafficking and endosomal sorting. This abnormality is similar to that observed in Dent’s disease, and it is of interest to note that some patients with the latter disease, who had no demonstrable CLC-5 mutations, were found instead to have *OCRL1* mutations [58]. The absence of cataracts in patients with Dent’s disease due to *OCRL1* mutations was the major phenotypic difference found when such patients were compared with patients with Lowe syndrome [58]. The molecular and cellular basis of these phenotypic differences still remains to be elucidated.

### Hereditary hypophosphatemic rickets with hypercalciuria

Two different heterozygous mutations (Ala48Phe and Val147Met) in NPT2a (also referred to as *SLC34A1*), the gene encoding a sodium-dependent phosphate transporter, have been reported in patients with urolithiasis or osteoporosis and persistent idiopathic hypophosphatemia due to decreased phosphate reabsorption by the renal tubules [64]. When expressed in *Xenopus laevis* oocytes, the mutant NPT2a showed impaired function. However, these in vitro findings were not confirmed in another study using oocytes and opossum kidney (OK) cells, raising the concern that the NPT2a mutation identified could not explain the findings in the patients described [65]. However, homozygous ablation of *Npt2a* in mice (*Npt2a-/-*) results in increased urinary phosphate excretion, hypophosphatemia, an appropriate elevation in the serum levels of 1,25-dihydroxyvitamin D, hypercalcemia, decreased serum parathyroid hormone levels, increased serum alkaline phosphatase activity and hypercalciuria (see below) [66]. Some of these biochemical features are observed in patients with hereditary hypophosphatemic rickets with hypercalciuria (HHRH), but there are important differences [67]. Thus, HHRH patients develop rickets, short stature, with an increased renal phosphate clearance, hypercalciuria, but have normal serum calcium levels, increased gastrointestinal absorption of calcium and phosphate due to an elevated serum concentration of 1,25-dihydroxyvitamin D, suppressed parathyroid function and normal urinary cAMP excretion [67]. However, HHRH patients do not have NPT2a mutations [68], and studies have demonstrated that HHRH patients harbor homozygous or compound heterozygous mutations of *SLC34A3*, the gene encoding the sodium–phosphate co-transporter NPT2c [69, 70]. These findings indicate that NPT2c has a more important role in phosphate homeostasis than was previously thought.

### Familial hypomagnesemia with hypercalciuria and nephrocalcinosis due to paracellin-1 (claudin 16) mutations

Familial hypomagnesemia with hypercalciuria and nephrocalcinosis (FHHNC) is an autosomal recessive renal tubular disorder that is frequently associated with progressive kidney failure [71]. FHHNC often presents in childhood, with seizures, or tetany due to hypocalcemia and hypomagnesemia. Other recurrent clinical manifestations include urinary tract infections, polyuria, polydipsia, and failure to thrive. Investigations reveal hypomagnesemia, hypocalcemia, hyperuricemia, hypermagnesuria, hypercalciuria, incomplete acidification in the distal renal tubules, hypocitraturia and renal calcification [72]. Treatment consists of high doses of magnesium, administered enterally, to restore normomagnesemia. Children with FHHNC who receive such treatment early, develop normally. Studies of linkage in 12 FHHNC kindreds found the disease locus to be on chromosome 3q27, and positional cloning studies identified mutations in the gene encoding paracellin-1 (PCLN-1), which is also referred to as claudin 16 (*CLDN16*) [72]. FHHNC patients were either homozygotes or compound heterozygotes for PCLN-1 mutations, consistent with the autosomal recessive inheritance of the disorder [73]. The PCLN-1 mutations consisted of premature termination codons, splice-site mutations and missense mutations [72–74]. The PCLN-1 protein, which consists of 305 amino acids, has sequence and structure similar to those of the members of the claudin family and is, therefore, also referred to as CLDN16 [72]. Claudins are membrane-bound proteins that form the intercellular tight junction barrier in a variety of epithelia [75]. Claudins have four transmembrane domains and intracellular amino- and carboxy-termini. The two luminal loops mediate cell–cell adhesion via homo- and hetero-typic interactions with claudins on a neighboring cell. In addition, claudins form paracellular ion channels, which facilitate renal tubular paracellular transport of solutes [75]. CLDN16 is exclusively expressed in the thick ascending limb of Henle’s loop, where it forms the paracellular channels that are driven by an electrochemical gradient and allow reabsorption of calcium and magnesium [76]. Hence, loss of function of CLDN16 that would arise from FHHNC mutations would result in urinary calcium and magnesium loss and lead to hypocalcemia and hypomagnesemia, respectively. A CLDN16 missense mutation (Thr233Arg) has also been identified in two families with self-limiting childhood hypercalciuria [74]. The hypercalciuria decreased with age and was not associated with progressive renal failure. The Thr233Arg mutation resulted in inactivation of a PDZ-domain binding motif, and this disrupted the association with the tight junction scaffolding protein, zona occludens (ZO)-1 [74], with accumulation of the mutant CLDN16 protein in lysosomes and no localization to the tight junctions. Thus, CLDN16 mutations may result in different abnormalities in the function of renal tubular cells and, hence, lead to differences in the clinical phenotype. A form of FHHNC with severe ocular involvement reported in one Swiss and eight Spanish/Hispanic families was recently mapped to chromosome 1p34.2 [77]. This region contains *CLDN19*, the gene that encodes claudin 19, a tight-junction protein expressed in the kidney and eye. A Gly20Asp mutation located in the first transmembrane domain of CLDN19 was identified in all but one of the Spanish/Hispanic families, and a Gln57Glu mutation in the first extracellular loop of CLDN19 was found in the Swiss family. In addition, a Leu90Pro mutation in CLDN19 was identified in a consanguineous family of Turkish origin with FHHNC and severe ocular involvement [77].

### Distal renal tubular acidosis

In distal renal tubular acidosis (dRTA) the tubular secretion of hydrogen ions in the distal nephron is impaired, and this results in a metabolic acidosis that is often associated with hypokalemia due to renal potassium wasting, hypercalciuria with nephrocalcinosis, and metabolic bone disease. Distal RTA may be familial, with autosomal dominant or recessive inheritance. One form of autosomal dominant dRTA is due to mutations of the erythrocyte anion exchanger (band 3, AE1). Two autosomal recessive forms of dRTA are caused by mutations of sub-units of the H^+^-ATPase (proton) pump. Thus, dRTA associated with sensorineural deafness is associated with mutations of the gene encoding the B1 subunit of the apical H^+^-ATPase pump (referred to as ATP6B1 or ATP6V1B1), whilst dRTA without deafness is caused by mutations of the gene encoding a different subunit, ATP6N1B (also referred to as ATP6V0A4), which is an isoform of ATP6N1A that is the 116 kDa non-catalytic accessory subunit of the proton pump.

#### Autosomal dominant dRTA due to erythrocyte anion exchanger (Band 3) mutations

The family of anion exchangers (AEs) is widely distributed and involved in the regulation of trans-cellular transport of acid and base across epithelial cells, cell volume and intracellular pH [78]. For example, AE1, which is a major glycoprotein of the erythrocyte membrane, mediates exchange of chloride and bicarbonate [79]. AE1 is also found in the basolateral membrane of the α-intercalated cells of renal collecting ducts, which are involved in acid secretion [80]. Patients with autosomal dominant dRTA, the majority of whom had hypercalciuria, renal stones and nephrocalcinosis and a few of them who had erythrocytosis, were found to have AE1 mutations [81]. These AE1 mutations resulted in several functional abnormalities that included reductions in chloride transport, and trafficking defects that led to cellular retention of AE1 or mis-targeting of AE1 to the apical membrane [82, 83]. AE1 mutations may also be associated with autosomal recessive dRTA in Southeast Asian kindreds that have ovalocytosis [84].

#### Autosomal recessive distal renal tubular acidosis due to proton pump mutations

Proton pumps (H^+^-ATPase) are ubiquitously expressed, and one such multi-unit H^+^-ATPase is found in abundance on the apical (luminal) surface of the α-intercalated cells of the cortical collecting duct, which regulates urinary acidification. Failure of vectorial proton transport by these α-intercalated cells results in the duct’s inability to acidify the urine and in disorders of dRTA. The molecular basis of two types of autosomal recessive dRTA due to proton pump abnormalities has been characterized. The gene causing one type of autosomal recessive dRTA that was associated with sensorineural hearing loss was mapped to chromosome 2p13, which contained the ATP6B1gene that encodes the B1 subunit of the apical proton pump (H^+^ATPase) [85]. Mutations that would likely result in a functional loss of ATP6B1 were identified in over 30% of families with this form of autosomal recessive dRTA that occurred with deafness in > 85% of families [85]. The association of dRTA and deafness is consistent with the renal and cochlear expression of ATP6B1 [86, 87]. ATP6B1 plays a critical role in regulating the pH of the inner ear’s endolymph, and dysfunction of this would lead to an alkaline micro-environment in the inner ear, which has been proposed to impair hair cell function and result in progressive deafness [85, 87]. The gene causing autosomal recessive dRTA with normal hearing was localized to chromosome 7q33-q34, which contained the ATP6N1B gene that encodes the non-catalytic accessory subunit of the proton pump of the α-intercalated cells of the collecting duct [88]. ATP6N1B mutations, which are predicted to result in a functional loss, were identified in > 85% of kindreds with autosomal recessive dRTA associated with normal hearing, and this is consistent with the expression of ATP6N1B in the kidney and not other organs. Approximately 15% of families with autosomal recessive dRTA were not found to have mutations in ATP6B1 or ATP6N1B mutations, and this indicates that mutations in other genes are likely to be involved in the etiology of autosomal recessive dRTA [89].

## Conclusions

**Fig. 1:**
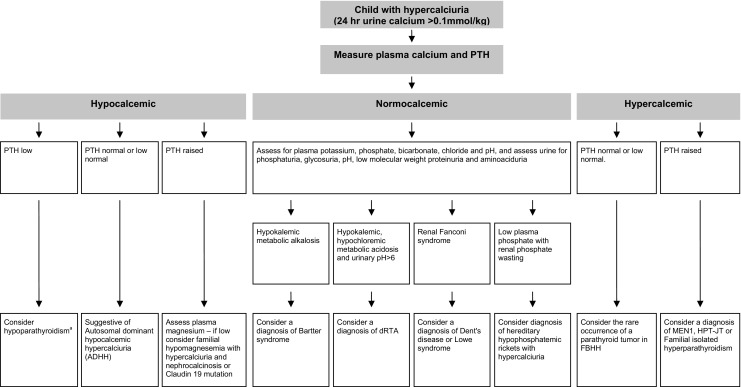
Suggested algorithm for the investigation of a child with hypercalciuria and nephrolithiasis/nephrocalcinosis, based initially on measurements of plasma calcium and PTH. For the hypocalcemic patient, hypoparathyroidism in this context, i.e. in association with hypercalciuria, is rare and is included here for completeness. One study of 85 patients with hypoparathyroidism and 15 ADHH patients has shown that prior to their treatment with vitamin D to correct the hypocalcemia, the urinary calcium/creatinine ratio in ADHH patients was generally higher than that found in patients with hypoparathyroidism, although there was an overlap [94]. Following treatment with vitamin D, the urinary calcium/creatinine ratios in ADHH and hypoparathyroid patients were similar [94]. Hence, it may be difficult for patients with hypoparathyroidism to be distinguished from those with ADHH on the sole basis of urinary calcium excretion evaluations [94]

Although kidney stones are an infrequent occurrence in children, they are, nevertheless, important causes of abdominal pain, urinary tract infection and progressive renal failure. Renal stones in children are associated with a metabolic abnormality in approximately 50% of patients, and treatment of this will help to prevent long-term complications. Thus, the investigation of hypercalciuric nephrolithiasis requires an assessment for these metabolic abnormalities, and a measurement of plasma and urinary electrolytes and metabolites is of importance. An approach to the diagnosis of these complex disorders, based upon the assessment of plasma calcium and PTH in a child with hypercalciuria, is outlined in Fig. 1. These metabolic disorders are often inherited and associated with hypercalciuria (Table 1), and studies of these disorders have elucidated some of the renal tubular transport mechanisms that regulate calcium reabsorption.

## Questions

### (Answers appear after the reference list)

1. Which of the following does NOT constitute a major risk factor for the development of kidney stones in children?
  - Anatomical abnormalities
  - Family history
  - Hypercalciuria
  - Urinary tract infection
  - Hypocalcemia
2. Which form of Bartter’s syndrome is NOT associated with nephrocalcinosis?
  - Type I
  - Type II
  - Type III
  - Type V
  - Type VI
3. The Bartter syndromes are characterized by which of the following clinical and biochemical features?
  - Hyperkalemia
  - Hypercalcemia
  - Metabolic alkalosis
  - It is always associated with an autosomal recessive mode of transmission
  - It may be associated with a defect in anion exchanger 1
4. Which of the following statements concerning idiopathic hypercalciuria (IH) is TRUE?
  - It is associated with 24-hour urinary calcium excretion of < 0.1mmol/kg
  - It may be associated with increased intestinal absorption of calcium
  - Families with IH often have recurrent calcium oxalate stones which have an autosomal recessive mode of inheritance
  - Mutations in the vitamin D receptor is a common cause of idiopathic hypercalciuria
5. Which of the following is NOT due to a mutation in the CaSR gene?
  - FIHP
  - Bartter’s syndrome
  - ADHH
  - Dent’s disease
  - FBHH
